# Editorial: Functional kinesiology in health and performance

**DOI:** 10.3389/fspor.2024.1428145

**Published:** 2024-05-22

**Authors:** Hadi Nobari, Antonio José Figueiredo, Kelly Johnson, Elena Mainer-Pardos

**Affiliations:** ^1^ Faculty of Sport Sciences, University of Extremadura, Cáceres, Spain; ^2^ Department of Exercise Physiology, Faculty of Educational Sciences and Psychology, University of Mohaghegh Ardabili, Ardabil, Iran; ^3^ Research Unit for Sport and Physical Activity, Faculty of Sport Sciences and Physical Education, University of Coimbra, Coimbra, Portugal; ^4^ Department of Kinesiology, Coastal Carolina University, Conway, SC, United States; ^5^ Health Sciences Faculty, Universidad San Jorge, Zaragoza, Spain

**Keywords:** anatomy, exercise, training, social health, strength

**Editorial on the Research Topic** Functional kinesiology in health and performance

## 1 Introduction

Functional kinesiology is a discipline that plays a crucial role in promoting optimal health and athletic performance. By combining biomechanics, anatomy, physiology, psychology, and neuroscience, it provides a comprehensive framework for understanding and enhancing human movement and function. In today's era of growing interest in health and athletic achievement, functional kinesiology represents an innovative approach that goes beyond traditional methods and transforms the way we study movement science. This editorial serves as an introduction to a series of articles that explore the various aspects of functional kinesiology and its impact on both health and performance.

## 2 Contributing articles

### 2.1 Exploring muscle synergies, loading effects, and training impact

This collection of articles explores different aspects of functional kinesiology, analyzing the complex relationship between muscle synergies, loading effects, and the impact of training on human movement and performance. Researchers use a combination of empirical studies and theoretical analyses to investigate the underlying mechanisms that govern motor control strategies, biomechanical adaptations, and physiological responses to exercise stimuli.

### 2.2 Insights into performance optimization and injury prevention

By analyzing various training methods, loading positions, and exercise interventions, these studies provide valuable insights into optimizing performance and preventing injuries (1). Researchers investigate the immediate and long-term effects of resistance training, endurance exercise, and neuromuscular conditioning on muscle function, body composition, functional asymmetries, joint stability, and movement efficiency. The results offer evidence-based guidelines for creating effective training programs tailored to individual needs and specific sports demands, ultimately improving athletic performance, and reducing the risk of musculoskeletal injuries (2, 3).

### 2.3 Implications for health, wellness, and long-term athletic development

Furthermore, this research contributes to our understanding of the wider impact of functional kinesiology on health, wellness, and long-term athletic development. Researchers are exploring the psychophysiological effects of training interventions, nutritional strategies, and recovery methods to enhance the physical and mental well-being of athletes over the course of their careers. The findings emphasize the significance of taking a holistic approach to training (4) and rehabilitation, which includes not only physical conditioning but also psychological or individual resilience (5), nutritional support, and lifestyle management.

### 2.4 Review of functional kinesiology in athletes

This section examines functional kinesiology research among male and female athletes of different age groups, with an emphasis on youth and adult categories. It integrates findings from various studies to provide an understanding of how kinesiological principles can enhance athletic performance and contribute to sustained health and well-being in competitive scenarios. This review highlights innovative approaches and emerging data linking functional kinesiology to improved athletic health and performance metrics.

### 2.5 Exploring the quality of training load and Bio-motor ability

This review focuses on examining studies that evaluate the quality of training load in athletes, specifically emphasizing bio-motor ability and wellness variables (6). By analyzing the relationship between training load metrics and performance outcomes, valuable insights are offered for optimizing training program design and workload management strategies to maximize athletic potential and minimize the risk of non-functional overreaching syndrome and injury (7).

## 3 Conclusion

In conclusion, this collection of articles provides a comprehensive exploration of functional kinesiology in health and performance contexts. Researchers have used a multidisciplinary approach to unravel the intricate mechanisms governing human movement and function, shedding light on the practical implications for optimizing athletic performance and promoting long-term health and well-being. These studies bridge the gap between theory and practice, paving the way for evidence-based interventions and strategies to empower athletes, coaches, and practitioners to achieve their full potential. Gratitude is extended to all the authors, reviewers, and contributors for enriching this collection with their valuable insights, and there is anticipation for further advancements in the field of functional kinesiology.
